# An 18‐month follow‐up of the Covid‐19 psychology research consortium study panel: Survey design and fieldwork procedures for Wave 6

**DOI:** 10.1002/mpr.1949

**Published:** 2022-10-10

**Authors:** Orla McBride, Sarah Butter, Anton P. Martinez, Mark Shevlin, Jamie Murphy, Todd K. Hartman, Ryan McKay, Philip Hyland, Kate M. Bennett, Thomas V. A. Stocks, Jilly Gibson‐Miller, Liat Levita, Liam Mason, Richard P. Bentall

**Affiliations:** ^1^ Ulster University Coleraine Northern Ireland; ^2^ University of Sheffield Sheffield UK; ^3^ University of Manchester Manchester UK; ^4^ Royal Holloway University of London Egham UK; ^5^ Maynooth University Maynooth Ireland; ^6^ University of Liverpool Liverpool UK; ^7^ University College London London UK

**Keywords:** attrition, COVID‐19, longitudinal survey, mental health, psychological

## Abstract

**Objectives:**

Established in March 2020, the C19PRC Study monitors the psychological and socio‐economic impact of the pandemic in the UK and other countries. This paper describes the protocol for Wave 6 (August–September 2021).

**Methods:**

The survey assessed: COVID‐19 related experiences; experiences of common mental health disorders; psychological characteristics; and social and political attitudes. Adult participants from any previous wave (*N* = 3170) were re‐invited, and sample replenishment procedures helped manage attrition. Weights were calculated using a survey raking algorithm to ensure the on‐going original panel (from baseline) was nationally representative in terms of gender, age, and household income, amongst other factors.

**Results:**

1643 adults were re‐interviewed at Wave 6 (51.8% retention rate). Non‐participation was higher younger adults, those born outside UK, and adults living in cities. Of the adults recruited at baseline, 54.3% (*N* = 1100) participated in Wave 6. New respondent (*N* = 415) entered the panel at this wave, resulting in cross‐sectional sample for Wave 6 of 2058 adults. The raking procedure re‐balanced the longitudinal panel to within 1.3% of population estimates for selected socio‐demographic characteristics.

**Conclusions:**

This paper outlines the growing strength of the publicly available C19PRC Study data for COVID‐19‐related interdisciplinary research.

## 1 INTRODUCTION

This is the sixth methodological report from the COVID‐19 Psychological Research Consortium (C19PRC) Study, a longitudinal, online survey of the UK adult population during the COVID‐19 pandemic which was funded by the UK Economic and Social Research Council. This report aims to provide important methodological information for secondary users of the C19PRC Study data, which is freely available via the Open Science Framework (OSF) (COVID‐19 Psychological Research Consortium, 2022). In the next section, we document the context for the sixth wave of the C19PRC Study during August–September 2021, before describing the design and conduct of the survey at this point in the pandemic.

### 1.1 Context for sixth wave of the C19PRC study

By summer 2021, the UK's hugely successful COVID‐19 vaccination programme had built a considerable ‘wall of defence’ against the coronavirus; approximately 49% of UK citizens aged 18 years and older were vaccinated with two doses of a COVID‐19 vaccine, and an additional 17% were partially vaccinated with one dose (Our World in Data, 2021). At that time, emerging evidence from Public Health England (2021) indicated that two doses of a vaccine provided superior protection compared to a single dose against the risk of symptomatic disease and hospitalisation as a result of infection with the now dominant, and highly transmissible, Delta variant. Thus, the UK Government further accelerated the vaccination programme during this period by reducing the interval between vaccine doses from 12 to 8 weeks for those under 40 years, to ensure that all adults aged 18 years and older who wanted to be fully vaccinated had the opportunity to do so by September 2021 (UK Government, 2021b). Young people aged 16–17 years were also being encouraged to receive their first vaccine dose before the start of the new academic year (Morton & Faulkner, 2021).

**FIGURE 1:**
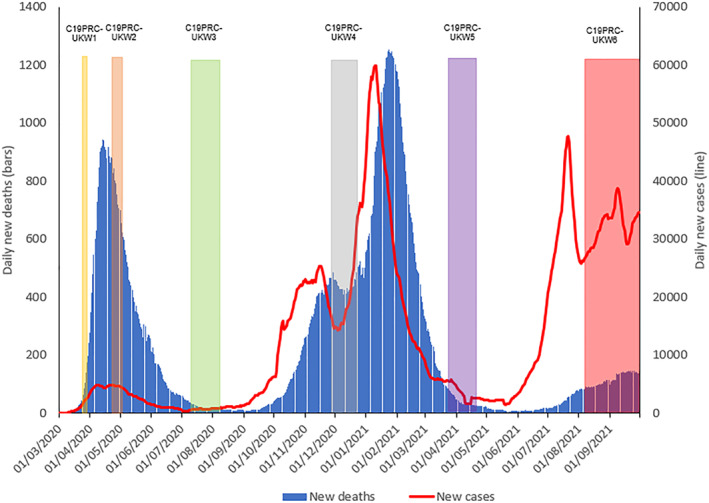
Graphical presentation of the number of daily COVID‐19 cases and deaths in the UK, sourced from Our World in Data (2021), aligned to the C19PRC Study survey waves. New daily deaths and cases depicted as 7 day rolling average

As was common throughout the pandemic, each UK nation imposed different regulations to manage the public health crisis (Institute for Government, 2022). In England during July 2021, the Government was working towards step 4 of ‘the roadmap’—this document, first published in February 2021, detailed step‐by‐step plans for the conditions that must be met to permit the relaxation of existing COVID‐19 regulations and public health guidance (UK Government, 2021c). Despite on‐going concerns about increasing rates of COVID‐19 infections and hospitalisations (Ball, 2021; Gurdasani et al., 2021) (see Figure 1), as well as ‘pandemic’ chaos caused by National Health Service (NHS) app notifications requiring hundreds of thousands of citizens to self‐isolate by law due to close contact with a positive case (James, 2021), July 19, 2021 was herald ‘Freedom Day’ in England. On this day, almost all of the public health guidance and restrictions that had been in place for approximately 15 months were lifted, including: the removal of social distancing measures and limits on social gatherings; mandatory use of face masks in shops and on public transport; work from home orders; and restrictions on operations of nightclubs, theatres, and restaurants (including the requirement for table service in hospitality). Somewhat contradictorily, the UK Government continued to promote a message of caution (UK Government, 2021e), urging: (i) citizens to exercise ‘personal judgment’ in their interactions with others and to meet up outside where possible; (ii) hospitality to continue with table service where possible; and (iii) employers to facilitate a gradual return to the workplace (UK Government, 2021a). Many shops continued to require the public to wear face masks and Transport for London ordered that face masks were a ‘condition of carriage’ (Menendez, 2021). Elsewhere, the devolved governments were more cautious, refusing to reopen fully at this time (Ball, 2021).

On July 1, 2021, it was announced that the UK Government's ‘Coronavirus Job Retention Scheme’ (CJRS) was set to end on September 30, 2021 (UK Government, 2021d). The CJRS was established in March 2020 to provide wages for employees who could not work due to the pandemic or to give financial support to employers to subside the wages of employees in work during the pandemic. Approximately 1.6 million people were covered by the CJRS (or ‘furloughed’) in July 2021, a substantial decrease from the 9 million working‐aged adults who were availing of the scheme in May 2020 (HM Revenue and Customs, 2021). However, the easing of restrictions resulted in a slower‐than‐anticipated growth of 1.3% in the UK's gross domestic product during quarter 3 of 2021 (July–September), but this was 2.1% below pre‐pandemic levels (i.e., of quarter 4, October–December 2019) (Office for National Statistics, 2021). As a result, over one million workers were expected to still require the CJRS when it was due to end in September 2021 (Tomlinson, 2021).

It was during this period of considerable change and uncertainty that planning for Wave 6 of the COVID‐19 Psychology Research Consortium (C19PRC) Study got underway. Launched in March 2020, the C19PRC is a longitudinal online survey which aims to monitor and assess the short‐to‐medium term psychological, social, economic, and political impacts of the COVID‐19 pandemic on the lives of UK adult citizens. Detailed methodological reports for previous waves are publicly available: Wave 1 (C19PRC‐UKW1; March 2020) and Wave 2 (C19PRC‐UKW2; April–May 2020) (McBride et al., 2021); Wave 3 (C19PRC‐UKW3; July‐August 2020) (McBride et al., 2021); Wave 4 (C19PRC‐UKW4; November–December 2020) (McBride et al., 2021); and Wave 5 (C19PRC‐UKW5; March–April 2021) (McBride et al., 2022).

The C19PRC Study's chief objective is to measure and explain changes (where evident) in the adult public's mental health and wellbeing, attitudes, beliefs, behaviours, and life experiences during the pandemic. To our knowledge, the C19PRC Study is one of the UK's longest running dedicated COVID‐19 surveys. A key feature of the C19PRC Study is the repeated assessment of *probable current* common mental disorders (major depression, generalised anxiety and COVID‐19 related posttraumatic stress disorder (PTSD)) using standardised and validated measures.

Our Consortium's ability to retain a sizeable proportion of the study's baseline sample over multiple waves, coupled with sample replenishment procedures at post‐baseline waves and targeted over‐sampling in the devolved UK nations, ensures that the C19PRC Study is well‐positioned to be an authoritative data source from which a public mental health evidence base on the pandemic can be produced (McBride et al., 2022).

The aim of this methodological report is two‐fold. First, we describe in detail the content of, and fieldwork for, this sixth survey wave (hereafter C19PRC‐UKW6) to stimulate its secondary use by interested researchers via OSF (COVID‐19 Psychological Research Consortium, 2022). Second, we report on: (i) attrition patterns in the C19PRC Study by the sixth wave, and whether these are associated with respondents' socio‐demographic characteristics at point of first entry to the study; (ii) sample replenishment and weighting procedures conducted to manage attrition in the longitudinal panel; and (iii) the socio‐demographic characteristics and prevalence of *probable current* common mental health problems (depression, anxiety, and COVID‐19 related PTSD) among participants in the C19PRC‐UKW6 sample.

## 2 METHOD

### 2.1 C19PRC‐UKW6: Fieldwork procedures

#### 2.1.1 Fieldwork organisation overview and strategy

As per previous waves, the marketing research company Qualtrics conducted the fieldwork for C19PRC‐UKW6 Qualtrics partners with over 20 online sample providers to supply a network of diverse, quality respondents to their worldwide client base. To date, the company has completed ∼15,000 projects across ∼2500 universities worldwide. Our Consortium has described in detail the quota‐based sampling strategy for the recruitment of the C19PRC Study baseline panel (McBride et al., 2021), as well as the replenishment of the panel during post‐baseline waves (McBride et al., 2022; McBride et al., 2021), via Qualtrics' traditional, actively managed, double‐opt‐in market research panels, which are used for corporate and academic market research only. We have also debated the strengths and limitations of the non‐probability sampling strategies we adopted to achieve the internet‐based, nationally representative C19PRC Study panel during an unprecedented time for survey fieldwork (McBride et al., 2021). We encourage interested readers to consult these reports for a more detailed interrogation of these methodological issues.

#### 2.1.2 Procedure

C19PRC‐UKW6 online fieldwork commenced on August 5, 2021, approximately 3 months after the completion of the fifth wave (C19PRC‐UKW5) and 15 months after the baseline survey (C19PRC‐UKW1) in March 2020. Two recruitment Phases were designed to achieve this aim (described next).

In Phase 1 (6 August to September 28, 2021), all adults (*N* = 3170) who commenced the survey at C19PRC‐UKW1 or joined the survey as a new entrant at either the third or fourth waves (C19PRC‐UKW3 or C19PRC‐UKW4) were eligible to be re‐contacted by Qualtrics and were invited to participate in this sixth wave. Qualtrics sent the following invitation via mail, SMS, or in‐app notifications to all eligible participants: ‘*Last year you participated in one or more surveys examining how the COVID‐19 pandemic is affecting the mental health and wellbeing of people in the UK. Your thoughts and opinions have been enormously valuable in helping us to understand the UK's experience of the pandemic. We would like to invite you to take part in a follow‐up survey, now that it has been more than 1 year since the pandemic began in the UK. This will help us create the most comprehensive picture ever achieved of how people cope and change during a global emergency. We hope you find it interesting*!’. No information was available as to whether any of the existing panel members had died over the course of the C19PRC Study between March 2020 and August‐September 2021.

During Phase 2 (8 to September 28, 2021), new participants were recruited from Qualtrics existing panels to fill quota gaps following the C19PRC‐UKW6 Phase 1 recruitment to ensure that the cross‐sectional sample for C19PRC‐UKW6 was nationally representative of the UK adult population with respect to age, gender, and household income.

As previously described (McBride et al., 2021), survey invitations were released in batches and, after the initial invitation was received, respondents who had not completed the survey were sent two reminders to encourage them to participate. The first reminder was sent approximately 36–48 h after the initial survey invite, with the second reminder sent another 36–48 h after this first reminder. As per previous waves (McBride et al., 2021), panel members were not obliged to participate in the sixth wave; however, they routinely receive an incentive for participation based on the length of the survey, their specific panellist profile, and target acquisition difficulty, amongst other factors. The specific type of reward varies and may include cash, air miles, gift cards, redeemable points, charitable donations, sweepstakes entrance, or vouchers (McBride et al., 2021).

#### 2.1.3 Informed consent process

At all survey waves, participants are informed that their data would be treated in confidence, that geolocating would be used to determine the area in which they lived (in conjunction with their residential postcode stem), and of their right to terminate participation at any time. Participants are also informed that some topics might be sensitive or distressing (e.g., self‐harm/suicide content). Information about how their data would be stored and analysed by the research team was also provided. Participants are also informed that they would be re‐contacted at a later date to invite them to participate in subsequent survey waves. Participants provided informed electronic consent prior to completing the survey and were directed to contact the NHS website upon completion if they had any concerns about COVID‐19, and emotional support services if they had been negatively impacted by any of the questions asked during the survey.

#### 2.1.4 Compliance with general data protection regulation

At all survey waves, participants are informed that C19PRC data will be stored confidentially in line with general data protection regulation. When the study data is deposited with the OSF, location data is removed and replaced with relevant socioeconomic summary data (e.g., area‐level deprivation and population density data). All other personal data is also removed.

#### 2.1.5 Quality control

As per previous waves (McBride et al., 2021), Qualtrics were charged with conducting multiple validation checks on the C19PRC survey data to identify and remove participants who (i) completed the survey faster than the pre‐set minimum completion time (to ensure responses were trustworthy); (ii) did not provide consent or meet eligibility criteria; or (iii) did not complete the survey in full. At this wave, 38 respondents from the existing panel were issued the survey as a pilot or ‘soft launch’ prior to the main fieldwork going live (‘full launch’) to rectify sequencing/coding errors and omissions. The median survey completion time for the wave is calculated to provide the survey team an opportunity to tailor the content to ensure the median survey time does not exceed 30 min. This is important to minimise respondent burden and maximise participation over time, as well as managing survey costs. The median completion time for the C19PRC‐UKW6 soft launch conducted during 5–6 August 2021 was 22 min 51 s. Only minor changes to the survey content were made following the soft launch and these participants were retained in the Phase 1 study sample.

### 2.2 Measures

An overview of the C19PRC‐UKW6 survey content is presented in Table 1.

**TABLE 1:** Overview of content of C19PRC Study Wave 6, UK, August–September 2021

COVID‐19 psychological research consortium study (C19PRC)—wave 6 (6 August–September 28, 2021)		
No. participants	*N* = 1643(R)	Details of validated measures
(R) = recontacts	*N* = 415(T)	
(T) = Top‐ups/new participants		
Sociodemographic		
Age	*x*	
Gender	*x*	
Ethnicity	*x*	
Region*	*x*	
Born in country of study	*x* ^*T*^	
Grow up in country of study	*x* ^*T*^	
Education level	*x*	
Religion	*x*	
Marital/relationship status	*x*	
Urbanicity of residence	*x*	
Economic activity (employment)	*x*	
Key/essential worker status	*x*	
Social class	*x*	
Social rank	*x*	
Sexual orientation	*x*	
Housing characteristics		
Number adults living in the home	*x*	
Number children living in the home	*x*	
Ages of children in the home	*x*	
Gender of children in the home	*x*	
Relationship to individuals living in the home	*x*	
Housing tenure	*x*	
Type of property	*x*	
Number of bedrooms	*x*	
Length of time at property	*x*	
Parental and children living in/out of the home status	*x*	
Pets in the home	*x*	
Household finances		
Estimated gross annual household income	*x*	
Change in monthly household income during pandemic	*x*	
Use of saving/increasing debt during pandemic	*x*	
Made saving due to pandemic	*x*	
Concern over household finances being negatively affected due to pandemic	*x*	
Perceived future financial security	*x*	
Receiving Government benefits	*x*	
Manageability of debt	*x*	
Difficulties paying bills	*x*	
Health conditions		
Major underlying health conditions ‐ self	*x* ^*T*^	
Major underlying health conditions ‐ immediate family member	*x* ^*T*^	
Currently pregnant ‐self/partner	*x*	
Number of weeks pregnant (if applicable)	*x*	
Currently pregnant–immediate family member	*x*	
Self‐rated health	*x*	
Family planning	*x*	
Children in the household		
Childcare for children in household during lockdown/summer holidays	*x*	
Perceived impact of the pandemic on child/children's wellbeing	*x*	
Warm and critical parenting behaviours	*x*	
Home‐schooling during summer months	*x*	
Parenting style	*x*	Parenting scale short form (PS‐8) (Kliem et al., 2019)
COVID‐19 impact on child education	*x*	
COVID‐19		
Anxiety level relating to COVID‐19	*x*	
Personal threat relating to COVID‐19	*x*	
Perceived individual risk of contracting COVID‐19 over next 6 months	*x*	
Perceived severity of symptoms	*x*	
Experiences of self‐isolation	*x*	
Experiences of children in the home self‐isolating	*x*	
Experiences of being infected with COVID‐19 (self and family member or friend)	*x*	
Experience of being tested for COVID‐19 (symptoms, location of testing/diagnosis)	*x*	
Experience of waiting to be tested for COVID‐19 (self)	*x*	
Knowing someone close (family member/friend) who has tested positive for COVID‐19	*x*	
Knowing someone close (family member/friend) who has died due to COVID‐19	*x*	
Social distancing/hygiene behaviour	*x*	
COVID‐19 vaccination status: Self	*x*	
COVID‐19 vaccine acceptability: Child	*x*	
COVID‐19 vaccine acceptability and hesitancy: Family & friends	*x*	
COVID‐19 booster vaccine acceptability: Self	*x*	
Reasons for accepting COVID‐19 vaccine: Self	*x*	
Reasons for refusing COVID‐19 vaccine: Self	*x*	
Reasons for refusing COVID‐19 vaccine: Child/children	*x*	
Reasons for deciding not to take second dose of vaccine	*x*	
Reasons for deciding not to take a booster vaccine	*x*	
Previous vaccine hesitancy during the pandemic	*x*	
Previous vaccine acceptability during the pandemic	*x*	
Reasons for changing mind about vaccine acceptability/hesitancy	*x*	
Non‐COVID vaccination status: Self	*x*	
Non‐COVID vaccination status of children	*x*	
Predicted course of the pandemic	*x*	
Perceptions of others' engagement in social distancing and health and safety guidance	*x*	
Support/opposition for end of COVID‐19 restrictions in England	*x*	
Mental health		
Depression (PHQ‐9)	x	PHQ‐9 (Kroenke, Spitzer, & Williams, 2001). ICD‐11 depression scale also included
Anxiety (GAD‐7)	*x*	GAD‐7 (Spitzer, Kroenke, Williams, & Löwe, 2006). ICD‐11 anxiety scale also included
Traumatic stress (ITQ)	*x*	ITQ (Cloitre et al., 2018)
Treatment for mental health difficulties	*x*	
Self‐harm, suicidal thoughts and attempts	*x*	
Post‐traumatic growth	*x*	Stress‐related growth scale revised [SRGS‐R]) (Boals & Schuler, 2018).
Psychotic experiences	*x*	Psychosis screening questionnaire, (Bebbington & Nayani, 1995)
Psychological factors		
Loneliness	*x*	3‐item loneliness scale (Hughes, Waite, Hawkley, & Cacioppo, 2004).
Self‐esteem	*x*	Single‐item self‐esteem scale (SISES) (Robins, Hendin, & Trzesniewski, 2001)
Resilience	*x*	Brief resilience scale (BRS) (Smith et al., 2008)
Attachment style	*x*	Experiences in close Relationships‐12 [ECR‐12] (Lafontaine et al., 2015)
Hopefulness	*x*	Brief‐H‐Positive scale (Fraser et al., 2014)
Happiness	*x*	1‐item happiness scale Spain: Pemberton happiness index
Life satisfaction (pre/post pandemic)	*x*	
Wellbeing	*x*	Warwick‐edinburgh mental wellbeing scale (WEMWBS, short 7‐item version) (Stewart‐Brown et al., 2009)
Benevolent childhood experiences	*x*	Benevolent Childhood experiences (BCE) scale (Narayan, Rivera, Bernstein, Harris & Lieberman, 2018).
Prisoner's dilemma game	*x*	
Numeracy test	*x*	3‐item basic numeracy test (Schwartz, Woloshin, Black, & Welch, 1997)
Wordsum vocabulary test	*x*	Wordsum test (Huang & Hauser, 1998)
Socio‐political views/related behaviour		
Voting behaviour at last general election	*x* ^*T*^	
Voting behaviour European referendum	*x* ^*T*^	
Measure of 'left wing'/'right‐wing' on socioeconomic issues	*x*	
Satisfaction with how government/institutions handling pandemic	*x*	
Patriotism/nationalism	*x*	
Child rearing views	*x*	
EU voter identification	*x*	
Impact of Brexit and pandemic on mental health	*x*	
Commonly debated political and social issues	*x*	
Trust		
Trust in other people (general)	*x*	
Facial detection of trust	*x*	

*X* ^*R*^ = Available for recontacted participants only.

*X* ^*T*^ = Available for top‐up/new participants only.

### 2.3 Ethical approval

The University of Sheffield provided ethical approval for the C19PRC Study (Reference number 033759).

### 2.4 Study variables

Given the broad focus of the C19PRC Study in understanding the impact of the pandemic on the UK adult general population, a wide range of socio‐demographic, economic, and psychological factors were selected to describe the characteristics of the sample participating at this wave, as well as to identify predictors of attrition from point of first entry into the study (i.e., C19PRC‐UKW1, C19PRC‐UKW3 or C19PRC‐UKW4) including: gender (females vs. males); age (18–24 years olds vs. 25–34 years, 35–44 years, 45–54 years, 55–64 years, and 65+ years groups); household income (≤£15,490 per annum vs. £15,491‐£25,340, £25,341‐£38,740, £38,741‐£57,903, and ≥£57,931 bands); economic activity (employed vs. other); ethnicity (White vs. other); born in UK (yes vs. no); household composition (living alone vs. other; children <18 years living in household vs. other); probable depression diagnosis (score of ≥10 on the *Patient Health Questionnaire‐9* vs. other); probable generalised anxiety diagnosis (score of ≥10 on the *Generalised Anxiety Disorder‐7* vs. other); probable PTSD diagnosis (using the *International Trauma Questionnaire's* diagnostic algorithm for PTSD caseness relating to experience of COVID‐19 vs. other); loneliness (score of ≥6 on the *Loneliness Scal*e); neuroticism (total score on the neuroticism subscale of the *Big‐Five Inventory‐10*); paranoia (total score on the *Persecution and Deservedness Scale*); and COVID‐19 anxiety (total score on single item indicator).

### 2.5 Data analysis plan and weighting procedures

Five sets of analyses are presented. First, re‐contact rates at C19PRC‐UKW6 were calculated for Phase 1. Second, a binary logistic regression model was conducted to assess the extent to which participation at C19PRC‐UKW6 could be predicted by a range of socio‐demographic and health‐related factors assessed at time the respondent first entered the C19PRC Study (i.e., at C19PRC‐UKW1, March 2020; C19PRC‐UKW3, July–August 2020; or C19PRC‐UKW4, November–December 2020). Third, the outcome of quota sampling at Phase 2 was determined by comparing the gender, age, and household income characteristics of the combined C19PRC‐UKW6 sample (i.e., Phases 1 and 2) to the specific sampling quotas set at baseline to obtain a nationally representative sample of UK adults. The percentage differences between the C19PRC‐UKW1 and C19PRC‐UKW6 quota bands for gender, age, and household income were calculated. Fourth, as per previous waves, raking procedures were conducted using the ‘anesrake’ package in *R* (Pasek & Pasek, 2018) to develop a survey weight to help account for attrition in the returning longitudinal panel (i.e., those adults entering the C19PRC Study at baseline participating in this sixth wave) with respect to the baseline proportions achieved for age, gender, household income, ethnicity, urbanicity, household composition (i.e., presence of children under 18 years), and being born or raised in the UK. This process has been described elsewhere (see McBride et al. (2021). And fifth, the current socio‐demographic and mental health characteristics of all adults who participated in C19PRC‐UKW6, having entered the study at different waves, were compared using cross‐tabulations.

## 3 RESULTS

### 3.1 Outcome of recruitment at Phase 1, C19PRC‐UKW6

At Phase 1, 3170 adults were eligible to participate in C19PRC‐UKW6. Contact with 1765 of eligible participants was established (55.7% recontact rate), but 122 (6.9%) of responses were deemed ineligible (and subsequently removed from the sample) due to: lack of completion of survey (64 respondents); not providing informed consent (50 respondents), and not meeting age eligibility criteria (8 respondents). In total, 1643 adults provided full interviews at Phase 1 (51.8% recontact rate).

The highest retention was from baseline respondents (C19PRC‐UKW1, March 2020), 54.3% (1100 respondents returned from eligible 2025), followed by those entering at the third wave (C19PRC‐UKW3; July 2020), 50.4% (430 respondents returned from an eligible 853) and then from those entering at the fourth wave (C19PRC‐UKW4; November 2020), 38.7% (113 respondents returned from an eligible 292). The median survey completion time for Phase 1 was 28 min 11 s.

### 3.2 Attrition at Phase 1, C19PRC‐UKW6

Table 2 presents the results of the binary logistic regression model predicting participation in C19PRC‐UKW6 from respondents' socio‐demographic, mental health, and psychological characteristics data collected at the first point of entry in the C19PRC Study. Compared to the youngest age group (18–24 years), adults in all other age groups were at higher odds of participating in this sixth wave (ORs ranged from 2.58 to 7.51). Adults living in cities were at lower odds of participating (OR 0.71; 95% CI 0.59–0.84) compared to those living in suburban, town or rural locations. Respondents born in the UK were at higher odds of participating in the sixth wave (OR 1.37; 95% CI 1.10–1.76) compared to those born elsewhere. Lower levels of paranoia predicted participation in this wave (OR = 0.98, 95% CI 0.96–0.99). Probable diagnoses of major depression, generalised anxiety disorder, or COVID‐19 related PTSD at point of entry to the study did not predict attrition at C19PRC‐UKW6.

**TABLE 2:** Adjusted odds ratios for characteristics of respondent participation at the sixth wave, C19PRC‐UKW6 August–September 2021

		Responder at C19PRC‐UKW6
Characteristics of respondent at point of entry to C19PRC study^a^		Odds ratio (95% confidence interval)
Gender^b^	Male	1
	Female	1.10 (0.94–1.30)
Age group (years)	18–24	1
	25–34	2.58 (1.97—3.38)***
	35–44	3.83 (2.88—5.10)***
	45–54	5.08 (3.80—6.78)***
	55–64	7.40 (5.40—10.16)***
	65+	7.51 (5.27—10.72)***
2019 household income	≤£15,490	1
	£15,491–£25,340	1.18 (0.91—1.53)
	£25,341–£38,740	1.10 (0.84—1.43)
	£38,741–£57,903	0.88 (0.68—1.15)
	≥£57,931	0.98 (0.75—1.30)
Employment	Other	1
	Employed	0.98 (0.81—1.19)
Born in the UK	No	1
	Yes	1.37 (1.10—1.76)*
Urbancity	Suburb/Town/Rural	1
	City	0.71 (0.59–0.84)***
Living alone	No	1
	Yes	1.14 (0.93—1.40)
Children in the household	No	1
	Yes	1.04 (0.88—1.23)
Chronic health condition	No	1
	Yes	1.11 (0.94–1.30)
Depression (PHQ‐9) caseness	No	1
	Yes	0.81 (0.64—1.04)
Anxiety (GAD‐7) caseness	No	1
	Yes	1.01 (0.79—1.30)
COVID‐19 PTSD caseness	No	1
	Yes	0.86 (0.68–1.09)
Loneliness caseness	No	1
	Yes	0.97 (0.81—1.16)
COVID‐19 anxiety		1.00 (0.99—1.00)
Neuroticism		1.05 (1.00–1.09)
Paranoia		0.98 (0.96—0.99)*

^a^ Point of entry includes baseline (C19PRC‐UKW1; March 2020); wave 3 (C19PRC‐UKW3; July‐August 2020) or wave 4 (C19PRC−UKW4; November−December 2020).

^b^ Participants classified as ‘Other gender’ not included due to low cell count.

*p < 0.05, ***p < 0.001.

### 3.3 Outcome of recruitment at Phase 1, C19PRC‐UKW6

Following an assessment of the characteristics of non‐respondents at Phase 1, Phase 2 ‘top‐up’ recruitment of new participants began according to key baseline sampling quotas for age, gender, and household income. Specifically, Phase 2 recruitment targeted women, younger adults (particularly those aged 18–34 years), and adults in low‐middle income brackets (£15,491‐£38,740)—see Table 3. The median completion time for the Phase 2 survey was 23 min 49 s. From a total of 601 eligible participants, quality control checks removed 186 respondents from Phase 2 due to: (i) participation in Phase 2 soft launch (50 respondents); (ii) breaching minimum survey completion time (34 respondents); (iii) not providing informed consent (47 respondents), (iv) not meeting age eligibility criteria (1 respondent); (v) not completing survey in full (22 respondents); (vi) previewing survey but withdrawing (2 respondents); (vii) attempting to take survey multiple times (28 participants); (viii) quota filled at the time survey participation was attempted (2 respondents). In total, 415 respondents provided full survey interviews at Phase 2.

**TABLE 3:** Outcome of quota sampling recruitment, COVID‐19 Psychological Research Consortium (C19PRC) Study UK Wave 6 (C19PRC‐UKW6), August–September 2021

Socio‐demographic characteristics used for quota sampling		Quotas C19PRC‐UKW1	C19PRC‐UKW6 Phase 1 (*N* = 1643)		C19PRC‐UKW6 Phase 2 ‘top‐up’ (*N* = 415)		C19PRC‐UKW6 sample (Phases 1 & Phase 2 ‘top‐up’) (*N* = 2058)		Percentage difference between quota target and quota obtained at C19PRC‐UKW6
		%	*n*	%	*n*	%	*n*	%	
Sex^a^	Men	49	794	48.3	189	45.5	983	47.8	−1.2
	Women	51	846	51.5	223	53.7	1069	51.9	+0.9
	Other	NA	3	0.2	3	0.7	6	0.3	NA
Age group (years)^a^	18–24	12	81	4.9	132	31.8	213	10.3	−1.7
	25–34	19	282	17.2	113	27.2	395	19.2	+0.2
	35–44	18	296	18.0	84	20.2	380	18.5	+0.5
	45–54	20	353	21.5	69	16.6	422	20.5	+0.5
	55–64	17	337	20.5	17	4.1	354	17.2	+0.2
	65+	14	294	17.9	−	−	294	14.3	+0.3
Gross annual household income^b^	£0–£15,490	20	315	19.2	81	19.5	396	19.2	−0.8
	£15,491–£25,340	20	291	17.7	99	23.9	390	19.0	−1.0
	£25,341–£38,740	20	321	19.5	104	25.1	425	20.7	+0.7
	£38,741–£57,930	20	349	21.2	71	17.1	420	20.4	+0.4
	£57,931+	20	367	22.3	60	14.5	427	20.7	+0.7

^a^ Quotas for age and sex were derived from EUROSTAT 2016 population estimates (Eurostat, 2020).

^b^ Quotas for gross household income bands were on 2016 Office for National Statistics data (Office for National Statistics, 2017).

**FIGURE 2:**
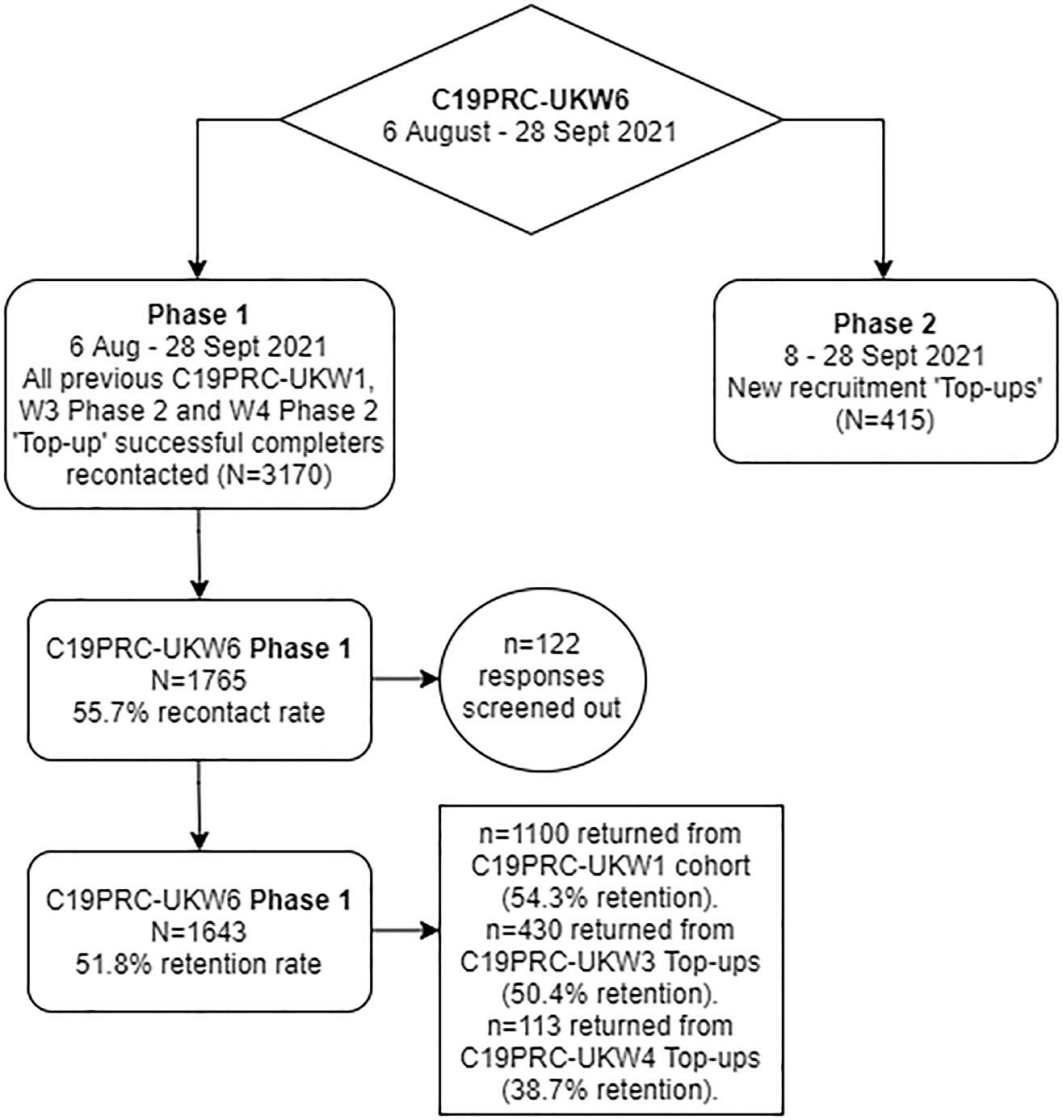
Flowchart of participation in the COVID‐19 Psychological Research Consortium Study (C19PRC) Study, Wave 6 (August–September 2021)

Overall, the combined cross‐sectional sample for C19PRC‐UKW6 (Phases 1 and 2; see Table 3) matched the established baseline sampling quotas for: gender (to within 0.9%; more women); age band (to within 1.7%; fewer adults aged 18–24 years); and annual household income (to within 1.0%; fewer adults earning £15,491‐£25,340). Figure 2 summarises the outcome of recruitment of C19PRC‐UKW6, Phase 1 and Phase 2.

### 3.4 Weighting procedure for longitudinal panel from baseline (C19PRC‐UKW1)

As presented in Supplementary Table S1, the raking procedure successfully re‐balanced the characteristics of responders at this sixth wave (*N* = 1100) to the baseline proportions for gender (rebalance within 1%), age (exact rebalance), household income (within 0.6%), household composition (exact rebalance), urbanicity (exact rebalance), ethnicity (within 0.9%), and born or raised in the UK (within 1.3%). Applying this weight for all analyses of the C19PRC‐UKW6 survey data completed by this longitudinal panel (who entered the panel at baseline) is recommended to account for attrition over survey waves on core study outcomes.

### 3.5 Socio‐demographic and mental health characteristics of adults participating in C19PRC‐UKW6

Table 4 presents cross‐tabulations for the socio‐demographic characteristics and the prevalence of *probable current* common mental health conditions of all adults who participated in C19PRC‐UKW6 (*N* = 2058), stratified by point of entry in the C19PRC Study. Statistically significant differences in characteristics between the C19PRC‐UKW6 respondents entering the C19PRC Study at different waves are indicated by different subscripts (see Table 4). Specifically, adults participating in this wave having entered the study at different time points varied with respect to age, household income, economic activity, ethnicity, presence of children living in household, and experiences of *probable current* mental health conditions.

**TABLE 4:** Socio‐demographic characteristics and prevalence of mental health disorders of the C19PRC‐UKW6 combined sample (*N* = 2058), stratified by phase of recruitment (August–September 2021)

Respondent characteristic		Phase 1—Longitudinal panel			Phase 2—New entrants	*χ* ^2^ (df), *p*
		From C19PRC‐UKW1 (*N* = 1100) *N* (%)	From C19PRC‐UKW3 (*N* = 430) *N* (%)	From C19PRC‐UKW4 (*N* = 113) *N* (%)	Quota top‐ups (*N* = 415) *N* (%)	
Gender	Male	550 (50.0)	198 (46.0)	52 (46.0)	189 (45.5)	3.18 (3), *p* > 0.05
	Female	550 (50.0)	231 (53.7)	60 (53.1)	223 (53.7)	
	Other categories^a^	−	1 (0.2)	1 (0.9)	3 (0.7)	
Age group (years)	18–24	63 (5.7)^a^	31 (7.2)^a^	20 (17.7)^b^	132 (31.8)^c^	390.19 (15), *p* < 0.001
	25–34	161 (14.6)^a^	103 (24.0)^b^	31 (27.4)^b^	113 (27.2)^b^	
	35–44	182 (16.5)^a^	105 (24.4)^b^	11 (9.7)^a^	84 (20.2)^a,b^	
	45–54	246 (22.4)^a^	88 (20.5)^a^	19 (16.8)^a^	69 (16.6)^a^	
	55–64	238 (21.6)^a^	67 (15.6)^b^	19 (16.8)^a,b^	17 (4.1)^c^	
	65+	210 (19.1)^a^	36 (8.4)^b^	13 (11.5)^a,b^	‐^c^	
2019 household income	£0–£15,490	231 (21.0)^a^	81 (18.8)^a^	19 (16.8)^a^	81 (19.5)^a^	30.85 (12), *p* < 0.01
	£15,491–£25,340	207 (18.8)^a^	81 (18.8)^a^	32 (28.3)^a^	99 (23.9)^a^	
	£25,341–£38,740	208 (18.9)^a^	95 (22.1)^a,b^	30 (26.5)^a,b^	104 (25.1)^b^	
	£38,741–£57,930	223 (20.3)^a^	90 (20.9)^a^	11 (9.7)^b^	71 (17.1)^a,b^	
	£57,931+	231 (21.0)^a^	83 (19.3)^a,b^	21 (18.6)^a,b^	60 (14.5)^b^	
Economic activity	(incl. Part‐time, self‐employed, zero‐hour contract, furlough)	677 (61.5)^a^	287 (66.7)^a,b^	75 (66.4)^a,b^	306 (73.7)^b^	20.34 (3), *p* < 0.001
	Other	423 (38.5)^a^	143 (33.3)^a,b^	38 (33.6)^a,b^	109 (26.3)^b^	
Ethnicity ^b^	White	1027 (93.4)^a^	379 (88.1)^b,c^	108 (95.6)^a,c^	356 (85.8)^b^	28.05 (3), *p* < 0.001
	Other	73 (6.6)^a^	51 (11.9)^b,c^	5 (4.4)^a,c^	59 (14.2)^b^	
Birthplace	Born in UK	1023 (93.0)	386 (89.8)	103 (91.2)	385 (92.8)	4.86 (3), *p* > 0.05
	Born elsewhere	77 (7.0)	44 (10.2)	10 (8.8)	30 (7.2)	
Household characteristics	Single adult household	233 (21.2)	91 (21.2)	33 (29.2)	99 (23.9)	4.80 (3), *p* > 0.05
	Other	867 (78.8)	339 (78.8)	80 (70.8)	316 (76.1)	
	Children under 18 years living in household	278 (25.3)^a^	138 (32.1)^b^	26 (23.0)^a,b^	189 (45.5)^c^	61.78 (3), *p* < 0.001
	Other	822 (74.7)^a^	292 (67.9)^b^	87 (77.0)^a,b^	226 (54.5)^c^	
Mental health conditions at W6	Depression–PHQ‐9 caseness	210 (19.1)^a^	92 (21.4)^a^	25 (22.1)^a^	198 (47.7)^b^	136.00 (3), *p* < 0.001
	Not met	890 (80.9)^a^	338 (78.6)^a^	88 (77.9)^a^	217 (52.3)^b^	
	Anxiety–GAD‐7 caseness	158 (14.4)^a^	77 (17.9)^a^	20 (17.7)^a^	170 (41.0)^b^	133.61 (3), *p* < 0.001
	Not met	942 (85.6)^a^	353 (82.1)^a^	93 (82.3)^a^	245 (59.0)^b^	
	PTSD caseness met	102 (9.3)^a^	38 (8.8)^a^	17 (15.0)^a^	158 (38.1)^b^	210.70 (3), *p* < 0.001
	Not met	998 (90.7)^a^	392 (91.2)^a^	96 (85.0)^a^	257 (61.9)^b^	

*Note*: ^abc^ Each subscript letter denotes a subset of recruitment phase whose column proportions do not differ from each other at the 0.05 level (Bonferroni adjusted).

^a^ 116 participants (Table 3)/5 participants (Table 4) classified as ‘Other gender’ not included due to low cell count.

^b^ All other variables use information collected at the Wave the participant first entered the survey—except ethnicity. Ethnicity was not collected at W3, so information used from W6 only here.

*p < 0.05, **p < 0.01, ***p < 0.001. C19PRC‐UKW1: baseline survey (March 2020).

For example, participants entering the C19PRC Study at post‐baseline waves were typically younger in age (e.g., 5.7% of those who participated in the sixth wave having entered at baseline were 18–24 years vs. 31.8% of those who entered the study at the sixth wave). This was to be expected due to sample replenishment procedures conducted to re‐balance the sample following attrition at this wave.

Fewer adults entering the C19PRC Study at post‐baseline waves were in the two highest household income brackets, compared to those who entered at baseline (e.g., 14.5% of adults first entering the survey at the sixth wave were earning £57,931+ per annum, compared to 21.0% of those entering at baseline). Higher proportions of adults entering the study post‐baseline and participating at this sixth wave were economically activity (e.g., 73.7% at C19PRC‐UKW6 compared to 61.5% at baseline), which likely reflects the improving economic environment in the UK following the easing of public health restrictions as the pandemic progressed, as well as the ‘top‐up’ procedures to target younger adults. Higher proportions of adults interviewed at this sixth wave who first entered the C19PRC study at the third or sixth waves were non‐white (11.9% and 14.2%, respectively) compared to baseline respondents who re‐interviewed at this sixth wave (6.6%). New entrants to the C19PRC Study at this sixth wave lived in households with children (45.5%) compared to those participating in this sixth wave who entered at baseline or the third or fourth waves (25.3%, 32.1% or 23.0%, respectively).

With respect to prevalence of caseness for *probable current* common mental health conditions, adults entering the C19PRC Study at this sixth wave had higher levels of *probable current* anxiety, depression, and COVID‐19 related PTSD (47.7%, 41.0%, and 38.1%, respectively) compared to adults who participated in this sixth wave having first entered the C19PRC Study at baseline or previous post‐baseline waves (i.e., *probable current* prevalence of anxiety, depression, and COVID‐19 related PTSD ≤ 22.1% for these respondent groups‐see Table 4).

## 4 DISCUSSION

During the first 18 months of the COVID‐19 pandemic in the UK, our Consortium established and successfully maintained a large, online survey to monitor and assess the psychological and social impact of the pandemic on the lives of the adult general population in the UK. Four main findings of this methodological report can be summarised succinctly. First, 51.8% of eligible adults in the panel were successfully re‐contacted and produced a full interview at this sixth survey wave. Second, non‐responders at this point in the study (August–September 2021) were characterised by being younger in age, born outside the UK, living in an urban area, and higher levels of paranoia; however, common mental health problems (e.g., anxiety and depression) at the point of entry into the study did not predict attrition at this wave. Third, targeted sample replenishment procedures were successful in: (i) boosting the cross‐sectional sample size to exceed two thousand participants at this wave, and (ii) rebalancing the sample characteristics to be representative of the UK adult general population in terms of age, gender, and household income. And fourth, the generation of survey weights for this sixth wave to ensure that all analyses of data produced by the original longitudinal panel (i.e., those recruited from baseline) accounts for respondent attrition during the C19PRC study was successful.

Comparing the performance of the C19PRC Study to other similar, existing studies is challenging due to the paucity of longitudinal studies or the lack of detailed methodological reports produced from similar studies with on‐going data collection 18 months into the pandemic. For example, the COVID‐19 Social Study is a long‐running online study in the UK which has a similar content and focus to the C19PRC Study. The COVID‐19 study started out as a weekly survey, but subsequently switched to monthly data collection approximately 5 months into the pandemic in August 2020, with new recruitment into the study halted at that time. The COVID‐19 Social Study, which comprises a well‐stratified but not random sample, similar to the C19PRC Study, conducted its 76th wave of data collection in late August/early September 2021. The study's technical report produced in September 2021 indicated that once the study ends, complete response rates for each wave will be calculated (Fancourt et al., 2021), which may help facilitate comparisons to the C19PRC Study metrics in the future. Elsewhere, the fourth wave of the Avon Longitudinal Study of Parents and Children (November 2020–March 2021), an existing, established cohort study which conducted a dedicated COVID‐19 survey, achieved a respectable 55% response rate (Smith et al., 2021), which is similar to what was achieved in this wave of the C19PRC Study. In terms of predictors of attrition, Yu et al. (2022) reported that younger age (18–24 years) predicted attrition across a three‐wave health‐related quality of life during COVID‐19 survey in the US between March–April 2020, July–September 2020, and January–March 2021; however, poorer mental health predicted attrition between the first two waves only, but not for the third wave conducted further into the pandemic. These findings are broadly consistent with our study's current and previous attrition analyses (McBride et al., 2021).

The COVID‐19 pandemic has forced a sizeable shift in how research is conducted. Governments across the world have relied on researchers to collect high‐quality, high frequency data from members of the general public to help assess and monitor changes in overall health, wellbeing, and socio‐economic circumstances to help guide and inform appropriate policy‐level responses to support ordinary citizens as they navigated life through the pandemic. The draconian restrictions on social interactions also necessitated the shift towards online survey‐based studies, due to the ability of this study design to collect data with greater ease and faster speed when compared to more traditional efforts (Haas et al., 2021; Singh & Sagar, 2021). The long‐term impact of the pandemic on the future of survey methodology has yet to be fully understood (Becker et al., 2022; Sattler et al., 2022) and may require novel approaches going forward (Yu et al., 2022).

Recent novel methodological work (Biddle & Sollis, 2021), however, has suggested that survey methodologists should consider routinely asking respondents about their subjective experiences of participating in COVID‐19 related (or similar) surveys. For example, in the Australian National University's five‐wave COVID‐19 Impact Monitoring Survey Program, answers to two questions (i.e., the respondent's subject experience as to how distressing the survey was, and how glad they were that they participated) were strong predictors of attrition over time—that is, those experiencing lower levels of distress while completing the survey and higher levels of gladness having completed the survey were more likely to complete subsequent waves (Biddle & Sollis, 2021). Elsewhere, Yu et al. (2022) reported that self‐report difficulty with the COVID‐19 health‐related quality of life survey predicted attrition across both follow‐up waves spanning the first year of the pandemic. Biddle and Sollis (2021) propose that this type of information about participants' subjective experience of survey participation is valuable for tailoring study invitation communications and/or incentives (where appropriate) in future waves in an attempt to minimise attrition in longitudinal panels. Our Consortium has one final wave planned for December 2022 (with two additional survey waves completed in November‐December 2021—Wave 7–May–June 2022—Wave 8–methodological reports forthcoming). To contribute to the limited field of research on survey participation during the COVID‐19 pandemic, we intend to ask panel members questions about their experiences of participation in our multi‐wave study with a view to shedding additional light on factors associated with full/partial participation across the C19PRC Study waves.

To conclude, our Consortium has carefully considered and debated the potential impact of the pandemic on our efforts to collect high‐quality, longitudinal data from a large sample of the UK adult general population (as well as ‘sister’ studies in other European countries) using non‐probability based sampling methods in detailed methodological reports from each wave of the C19PRC Study (McBride et al., 2022; McBride et al., 2022). As per best practice (Besançon et al., 2021), we have been strongly committed to Open Science principles from the outset. We encourage interested readers to consult our detailed methodological reports and to access the data via the OSF for exploitation and secondary use purposes when the broad and deep coverage of the C19PRC Study survey data may be suitable for addressing important research questions of public health interest.

## CONFLICT OF INTEREST

All authors declare no conflict of interest.

## ETHICS STATEMENT

The University of Sheffield provided ethical approval for the C19PRC Study (Reference number 033759).

## Supporting information

Supplementary Material 1

## Data Availability

The data and associated documentation related to C19PRC‐UKW1—C19PRC‐UKW6 are publicly available via the Open Science Framework (see https://osf.io/v2zur/files/).

## References

[mpr1949-bib-0001] Ball, P. (2021). Why England’s Covid freedom day alarms researchers. Nature, 595(7868), 479–480. 10.1038/d41586-021-01938-4 34262196

[mpr1949-bib-0035] Bebbington, P. , & Nayani, T. (1995). The Psychosis Screening Questionnaire. International Journal of Methods in Psychiatric Research, 5, 1 ‐19.

[mpr1949-bib-0002] Becker, R. , Möser, S. , Moser, N. , & Glauser, D. (2022). Survey participation in the long‐lasting time of Corona: A replication of the Covid‐19‐pandemic effect on survey participation. Retrieved from https://www.edu.unibe.ch/unibe/portal/fak_humanwis/philhum_institute/inst_paed/content/e66/e507334/e650306/pane652747/e1204020/SurveyParticipationintheLong‐lastingTimeofCoronafv16‐March‐2022_ger.pdf

[mpr1949-bib-0003] Besançon, L. , Peiffer‐Smadja, N. , Segalas, C. , Jiang, H. , Masuzzo, P. , Smout, C. , Billy, E. , Deforet, M. , & Leyrat, C. (2021). Open science saves lives: Lessons from the Covid‐19 pandemic. BMC Medical Research Methodology, 21(1), 1–18. 10.1186/s12874-021-01304-y 34090351 PMC8179078

[mpr1949-bib-0004] Biddle, N. , & Sollis, K. (2021). Determinants of participation in a longitudinal survey during the Covid‐19 pandemic: The case of a low‐infection Country. Retrieved from https://openresearch‐repository.anu.edu.au/bitstream/1885/261422/1/Determinants_of_participation_in_a_longitudinal_survey_during_the_COVID‐19_pandemic_‐_Biddle_and_Sollis_2021_‐_For_web.pdf 10.1332/175795921X16730110266038 37022324

[mpr1949-bib-0036] Boals, A. , & Schuler, K. L. (2018). Reducing Reports of Illusory Posttraumatic Growth: A Revised Version of the Stress‐Related Growth Scale (Srgs‐R). Psychological Trauma: Theory, Research, Practice, and Policy, 10(2), 190. 10.1037/tra0000267 28368153

[mpr1949-bib-0037] Cloitre, M. , Shevlin, M. , Brewin, C. R. , Bisson, J. I. , Roberts, N. P. , Maercker, A. , Karatzias, T. , & Hyland, P. (2018). The International Trauma Questionnaire: Development of a Self‐Report Measure of Icd‐11 Ptsd and Complex Ptsd. Acta Psychiatrica Scandinavica, 138(6), 536‐546. 10.1111/acps.12956 30178492

[mpr1949-bib-0005] COVID‐19 Psychological Research Consortium . (2022). Covid‐19 psychological research Consortium (C19prc) study (2020–2022). 10.17605/OSF.IO/V2ZUR

[mpr1949-bib-0006] Eurostat . (2020). Population on 1 January by age and sex. Retrieved from https://appsso.eurostat.ec.europa.eu/nui/show.do?dataset=demo_pjan%26lang=en

[mpr1949-bib-0007] Fancourt, D. , Paul, E. , & Feifer, B. (2021). Covid‐19 social study: Data user guide version 38. Retrieved from https://osf.io/jwgt7/

[mpr1949-bib-0038] Fraser, L. , Burnell, M. , Salter, L. C. , Fourkala, E.‐O. , Kalsi, J. , Ryan, A. , Gessler, S. , Gidron, Y. , Steptoe, A. , & Menon, U. (2014). Identifying Hopelessness in Population Research: A Validation Study of Two Brief Measures of Hopelessness. BMJ Open, 4(5). 10.1136/bmjopen-2014-005093 PMC4039863 24879829

[mpr1949-bib-0008] Gurdasani, D. , Drury, J. , Greenhalgh, T. , Griffin, S. , Haque, Z. , Hyde, Z. , Katzourakis, A. , McKee, M. , Michie, S. , Pagel, C. , Reicher, S. , Roberts, A. , West, R. , Yates, C. , & Ziauddeen, H. (2021). Mass infection is not an option: We must do more to protect our young. The Lancet, 398(10297), 297–298. 10.1016/S0140-6736(21)01589-0 PMC8262842 34245669

[mpr1949-bib-0009] Haas, G.‐C. , Müller, B. , Osiander, C. , Schmidtke, J. , Trahms, A. , Volkert, M. , & Zins, S. (2021). Development of a new Covid‐19 panel survey: The IAB high‐frequency online personal panel (HOPP). Journal for Labour Market Research, 55(1), 1–14. 10.1186/s12651-021-00295-z 34179683 PMC8220878

[mpr1949-bib-0010] HM Revenue and Customs . (2021). Official Statistics. Coronavirus Job retention scheme Statistics: 9 September 2021. Retrieved from https://www.gov.uk/government/statistics/coronavirus‐job‐retention‐scheme‐statistics‐9‐september‐2021/coronavirus‐job‐retention‐scheme‐statistics‐9‐september‐2021

[mpr1949-bib-0039] Huang, M.‐H. , & Hauser, R. (1998). Trends in Black‐White Test‐Score Differentials: II. The Wordsum Vocabulary Test. American Psychological Association.

[mpr1949-bib-0040] Hughes, M. E. , Waite, L. J. , Hawkley, L. C. , & Cacioppo, J. T. (2004). A Short Scale for Measuring Loneliness in Large Surveys: Results from Two Population‐Based Studies. Research on Aging, 26(6), 655‐672. 10.1177/0164027504268574 18504506 PMC2394670

[mpr1949-bib-0011] Institute for Government . (2022). Coronavirus lockdown rules in each part of the UK. Retrieved from https://www.instituteforgovernment.org.uk/explainers/coronavirus‐lockdown‐rules‐four‐nations‐uk?msclkid=ba9b5906b57911ec99a7487dfdec853d

[mpr1949-bib-0012] James, W. (2021). England's 'freedom day' marred by soaring cases and isolation chaos. Reuters. Retrieved from https://www.reuters.com/world/uk/pm‐johnson‐pleads‐caution‐freedom‐day‐arrives‐england‐2021‐07‐18/

[mpr1949-bib-0041] Kliem, S. , Lohmann, A. , Mößle, T. , Foran, H. M. , Hahlweg, K. , Zenger, M. , & Brähler, E. (2019). Development and Validation of a Parenting Scale Short Form (Ps‐8) in a Representative Population Sample. Journal of Child and Family Studies, 28(1), 30‐41. 10.1007/s10826-018-1257-3

[mpr1949-bib-0042] Kroenke, K. , Spitzer, R. L. , & Williams, J. B. (2001). The Phq‐9: Validity of a Brief Depression Severity Measure. Journal of General Internal Medicine, 16(9), 606‐613. 10.1046/j.1525-1497.2001.016009606.x 11556941 PMC1495268

[mpr1949-bib-0043] Lafontaine, M.‐F. , Brassard, A. , Lussier, Y. , Valois, P. , Shaver, P. R. , & Johnson, S. M. (2015). Selecting the Best Items for a Short‐Form of the Experiences in Close Relationships Questionnaire. European Journal of Psychological Assessment, 32(2). 10.1037/tra0000267

[mpr1949-bib-0013] McBride, O. , Butter, S. , Hartman, T. K. , Murphy, J. , Hyland, P. , Shevlin, M. , Gibson‐Miller, J. , Levita, L. , Mason, L. , Martinez, A. P. , McKay, R. , Lloyd, A. , Stocks, T. V. A. , Bennett, J. , Vallières, F. , Karatzias, T. , Valiente, C. , Vazquez, C. , Contreras, A. , … Bentall, R. P. (2022). Sharing data to better understand one of the world’s most significant shared experiences: Data resource profile of the longitudinal Covid‐19 psychological research Consortium (C19prc) study. International Journal of Population Data Science, 5(4). 10.23889/ijpds.v5i4.1704 PMC8900652 35310464

[mpr1949-bib-0014] McBride, O. , Butter, S. , Murphy, J. , Hartman, T. K. , McKay, R. , Shevlin, M. , Bennett, K. , Stocks, T. V. , Lloyd, A. , Gibson‐Miller, J. , Levita, L. , Mason, L. , Martinez, A. P. , Vallieres, F. , Karatzias, T. , & Bentall, R. P. (2022). Tracking the psychological and socio‐economic impact of the Covid‐19 pandemic in the UK: A methodological report from wave 5 of the Covid‐19 psychological research Consortium (C19PRC) study. International Journal of Methods in Psychiatric Research. 10.1002/mpr.1928 PMC9349513 35759532

[mpr1949-bib-0015] McBride, O. , Butter, S. , Murphy, J. , Shevlin, M. , Hartman, T. K. , Bennett, K. , Stocks, T. V. , Lloyd, A. , McKay, R. , Gibson‐Miller, J. , Levita, L. , Mason, L. , Martinez, A. P. , Hyland, P. , Vallieres, F. , Karatzias, T. , Valiente, C. , Vazquez, C. , & Bentall, R. P. (2021). Design, content, and fieldwork procedures of the Covid‐19 psychological research Consortium (C19prc) study—Wave 4. International Journal of Methods in Psychiatric Research, 31(1). 10.1002/mpr.1899 PMC8646695 34739156

[mpr1949-bib-0016] McBride, O. , Butter, S. , Murphy, J. , Shevlin, M. , Hartman, T. K. , Hyland, P. , McKay, R. , Bennett, K. , Gibson‐Miller, J. , Levita, L. , Mason, L. , Martinez, A. P. , Stocks, T. V. , Vallieres, F. , Karatzias, T. , Valiente, C. , Vazquez, C. , & Bentall, R. P. (2021). Context, design and conduct of the longitudinal Covid‐19 psychological research Consortium (C19prc) study—Wave 3. International Journal of Methods in Psychiatric Research, 30, 1–17. 10.1002/mpr.1880 PMC8209941 34021946

[mpr1949-bib-0017] McBride, O. , Murphy, J. , Shevlin, M. , Gibson‐Miller, J. , Hartman, T. K. , Hyland, P. , Levita, L. , Mason, L. , Martinez, A. P. , McKay, R. , Stocks, T. V. , Bennett, K. , Vallières, F. , Karatzias, T. , Valiente, C. , Vazquez, C. , & Bentall, R. P. (2021). Monitoring the psychological, social, and economic impact of the Covid‐19 pandemic in the population: Context, design and conduct of the longitudinal Covid‐19 psychological research Consortium (C19PRC) study. International Journal of Methods in Psychiatric Research, 30(1), 1–16. 10.1002/mpr.1861 PMC7992290 33166018

[mpr1949-bib-0018] Menendez, E. (2021). Face masks to remain compulsory on London buses and tube despite freedom day. Metro. Retrieved from https://metro.co.uk/2021/07/13/face‐masks‐to‐remain‐compulsory‐on‐london‐buses‐and‐tube‐despite‐freedom‐day‐14924735/

[mpr1949-bib-0019] Morton, B. , & Faulkner, D. (2021). Covid: First 16 and 17‐year‐olds begin to get vaccine invites. BBC. Retrieved from https://www.bbc.com/news/uk‐58112765?msclkid=1c01ac2fb57911ecbf5416b12110f219

[mpr1949-bib-0044] Narayan, A. J. , Rivera, L. M. , Bernstein, R. E. , Harris, W. W. , & Lieberman, A. F. (2018). Positive Childhood Experiences Predict Less Psychopathology and Stress in Pregnant Women with Childhood Adversity: A Pilot Study of the Benevolent Childhood Experiences (Bces) Scale. Child Abuse and Neglect, 78, 19‐30. 10.1016/j.chiabu.2017.09.022 28992958

[mpr1949-bib-0020] Office for National Statistics . (2017). Household disposable income and inequality in the UK: Financial year ending 2016. Retrieved from https://www.ons.gov.uk/peoplepopulationandcommunity/personalandhouseholdfinances/incomeandwealth/bulletins/householddisposableincomeandinequality/financialyearending2016

[mpr1949-bib-0021] Office for National Statistics . (2021). GDP quarterly national accounts. Retrieved from https://www.ons.gov.uk/economy/grossdomesticproductgdp/bulletins/quarterlynationalaccounts/julytoseptember2021

[mpr1949-bib-0022] Our World in Data . (2021). Share of people vaccinated against Covid‐19. https://ourworldindata.org/explorers/coronavirus‐data‐explorer?zoomToSelection=true%26time=2021‐07‐02%26facet=none%26pickerSort=asc%26pickerMetric=location%26Metric=People+vaccinated+%28by+dose%29%26Interval=7‐day+rolling+average%26Relative+to+Population=true%26Color+by+test+positivity=false%26country=~GBR

[mpr1949-bib-0045] Pasek, J. , & Pasek, M. (2018). Anes Raking Implementation and Weighted Statistics. Retrieved from https://cran.r‐project.org/web/packages/anesrake/anesrake.pdf

[mpr1949-bib-0023] Public Health England . (2021). Vaccines highly effective against B.1.617.2 variant after 2 doses. Retrieved from https://www.gov.uk/government/news/vaccines‐highly‐effective‐against‐b‐1‐617‐2‐variant‐after‐2‐doses?msclkid=7f50d2a2b57811eca0f98fb52eefd510

[mpr1949-bib-0046] Robins, R. W. , Hendin, H. M. , & Trzesniewski, K. H. (2001). Measuring Global Self‐Esteem: Construct Validation of a Single‐Item Measure and the Rosenberg Self‐Esteem Scale. Personality and Social Psychology Bulletin, 27(2), 151‐161.

[mpr1949-bib-0024] Sattler, C. , Rommel, J. , Chen, C. , García‐Llorente, M. , Gutiérrez‐Briceño, I. , Prager, K. , Reyes, M. F. , Schröter, B. , Schulze, C. , van Bussel, L. G. , Loft, L. , Matzdorf, B. , & Kelemen, E. (2022). Participatory research in times of Covid‐19 and beyond: Adjusting your methodological toolkits. One Earth, 5(1), 62‐73. 10.1016/j.oneear.2021.12.006 35098107 PMC8779601

[mpr1949-bib-0047] Schwartz, L. M. , Woloshin, S. , Black, W. C. , & Welch, H. G. (1997). The Role of Numeracy in Understanding the Benefit of Screening Mammography. Annals of internal medicine, 127(11), 966‐972. 10.7326/0003-4819-127-11-199712010-00003 9412301

[mpr1949-bib-0025] Singh, S. , & Sagar, R. (2021). A critical look at online survey or questionnaire‐based research studies during Covid‐19. Asian Journal of Psychiatry, 65, 102850. 10.1016/j.ajp.2021.102850 34534919 PMC8426323

[mpr1949-bib-0050] Smith, B. W. , Dalen, J. , Wiggins, K. , Tooley, E. , Christopher, P. , & Bernard, J. (2008). The Brief Resilience Scale: Assessing the Ability to Bounce Back. International Journal of Behavioral Medicine, 15(3), 194‐200. 10.1080/10705500802222972 18696313

[mpr1949-bib-0026] Smith, D. , Bowring, C. , Wells, N. , Crawford, M. , Timpson, N. J. , & Northstone, K. (2021). The Avon longitudinal study of Parents and children‐a resource for Covid‐19 research: Questionnaire data capture November 2020–March 2021. Wellcome Open Research, 6, 155. 10.12688/wellcomeopenres.16950.2 34796274 PMC8591520

[mpr1949-bib-0048] Spitzer, R. L. , Kroenke, K. , Williams, J. B. , & Löwe, B. (2006). A Brief Measure for Assessing Generalized Anxiety Disorder: The Gad‐7. Archives of Internal Medicine, 166(10), 1092‐1097. http://10.1001/archinte.166.10.1092 16717171 10.1001/archinte.166.10.1092

[mpr1949-bib-0049] Stewart‐Brown, S. , Tennant, A. , Tennant, R. , Platt, S. , Parkinson, J. , & Weich, S. (2009). Internal Construct Validity of the Warwick‐Edinburgh Mental Well‐Being Scale (Wemwbs): A Rasch Analysis Using Data from the Scottish Health Education Population Survey. Health and Quality of Life Outcomes, 7(1), 1‐8. 10.1186/1477-7525-7-15 19228398 PMC2669062

[mpr1949-bib-0027] Tomlinson, D. (2021). Job well done: 18 Months of the coronavirus Job retention scheme. Resolution Foundation. Retrieved from https://www.resolutionfoundation.org/publications/job‐well‐done/

[mpr1949-bib-0028] UK Government . (2021a). Orla statement to parliament: Secretary of state for health and social care provides an update on step 4. Retrieved from https://www.gov.uk/government/speeches/secretary‐of‐state‐for‐health‐and‐social‐care‐provides‐an‐update‐on‐step‐4

[mpr1949-bib-0029] UK Government . (2021b). Covid‐19 response: Summer 2021. Retrieved from https://assets.publishing.service.gov.uk/government/uploads/system/uploads/attachment_data/file/999419/COVID‐19_Response_Summer_2021.pdf?msclkid=4a676b01b57811ecbd7ea2d0ba3ce262

[mpr1949-bib-0030] UK Government . (2021c). Guidance: Moving to step 4 of the roadmap. Retrieved from https://www.gov.uk/government/publications/covid‐19‐response‐summer‐2021‐roadmap/moving‐to‐step‐4‐of‐the‐roadmap?msclkid=12ddb612b57a11ec9b3877735ea86c73

[mpr1949-bib-0031] UK Government . (2021d). Policy paper: Changes to the coronavirus Job retention scheme from July 2021. Retrieved from https://www.gov.uk/government/publications/changes‐to‐the‐coronavirus‐job‐retention‐scheme/changes‐to‐the‐coronavirus‐job‐retention‐scheme

[mpr1949-bib-0032] UK Government . (2021e). Speech: Pm statement at coronavirus press conference: 12 July 2021. Retrieved from https://www.gov.uk/government/speeches/pm‐statement‐at‐coronavirus‐press‐conference‐12‐july‐2021

[mpr1949-bib-0033] Yu, T. , Chen, J. , Gu, N. Y. , Hay, J. W. , & Gong, C. L. (2022). Predicting panel attrition in longitudinal HRQOL surveys during the Covid‐19 pandemic in the US. Health and Quality of Life Outcomes, 20(1), 1‐12. 10.1186/s12955-022-02015-8 35794553 PMC9258760

