# Supplementary material for: An 18‐month follow‐up of the Covid‐19 psychology research consortium study panel: Survey design and fieldwork procedures for Wave 6
Source: Int J Methods Psychiatr Res. 2022 Oct 10;32(2):e1949. doi: 10.1002/mpr.1949 (PMC9874753; doi:10.1002/mpr.1949)
Supplement: Supplementary file 1 — Supplementary Material 1 [file MPR-32-e1949-s001.docx]

Supplementary Table 1 Outcome of raking weighting procedure at C19PRC UK Wave 6, August-September 2021 (N=1100)

| **W1 variables used for weighting** | Wave 1 (N=2025) | Wave 6 recontacts* (not weighted; N=1100) | W6 recontacts* (weighted – 5% weight; N=1100) |
| --- | --- | --- | --- |
| **Age** |  |  |  |
| 18-24 | 246 (12.1%) | 63 (5.7%) | 134 (12.1%) |
| 25-34 | 380 (18.8%) | 161 (14.6%) | 206 (18.8%) |
| 35-44 | 353 (17.4%) | 182 (16.5%) | 192 (17.4%) |
| 45-54 | 410 (20.2%) | 246 (22.4%) | 223 (20.2%) |
| 55-64 | 349 (17.2%) | 238 (21.6%) | 190 (17.2%) |
| 65+ | 287 (14.2%) | 210 (19.1%) | 156 (14.2%) |
| **Gender** |  |  |  |
| Male | 972 (48.0%) | 550 (50.0%) | 516 (46.9%) |
| Female | 1053 (52.0%) | 550 (50.0%) | 584 (53.1%) |
| **Income** |  |  |  |
| £0-300 per week | 410 (20.2%) | 231 (21.0%) | 229 (20.8%) |
| £301-490 per week | 410 (20.2%) | 207 (18.8%) | 225 (20.4%) |
| £491-740 per week | 385 (19.0%) | 208 (18.9%) | 209 (19.0%) |
| £741-1,111 per week | 410 (20.2%) | 223 (20.3%) | 216 (19.6%) |
| £1,112 or more per week | 410 (20.2%) | 231 (21.0%) | 221 (20.1%) |
| **Urbanicity** |  |  |  |
| City | 498 (24.6%) | 214 (19.5%) | 271 (24.6%) |
| Suburb/Town/Rural | 1527 (75.4%) | 886 (80.5%) | 829 (75.4%) |
| **Ethnicity** |  |  |  |
| White | 1848 (91.3%) | 1030 (93.6%) | 1014 (92.2%) |
| Non-white | 177 (8.7%) | 70 (6.4%) | 86 (7.8%) |
| **Household composition** |  |  |  |
| Children in household | 592 (29.2%) | 278 (25.3%) | 322 (29.2%) |
| No children in household | 1433 (70.8%) | 822 (74.7%) | 778 (70.8%) |
| **Born or raised in UK** |  |  |  |
| Yes | 1891 (93.4%) | 1044 (94.9%) | 1042 (94.7%) |
| No | 134 (6.6%) | 56 (5.1%) | 58 (5.3%) |
|  |  |  |  |

*Recontacted from Wave 1 only, not from Wave 3 top-ups or Wave 4 top-ups or boosters.

Other gender categories combined with female for purposes of weighting.

Correlation between 5% and 1% weights = 0.975
